# Dietary partitioning promotes the coexistence of planktivorous species on coral reefs

**DOI:** 10.1111/mec.15090

**Published:** 2019-05-13

**Authors:** Matthieu Leray, Alice L. Alldredge, Joy Y. Yang, Christopher P. Meyer, Sally J. Holbrook, Russell J. Schmitt, Nancy Knowlton, Andrew J. Brooks

**Affiliations:** ^1^ Smithsonian Tropical Research Institute, Smithsonian Institution Panama City, Balboa, Ancon Panama; ^2^ Department of Ecology, Evolution and Marine Biology University of California Santa Barbara Santa Barbara California; ^3^ Computational and Systems Biology Massachusetts Institute of Technology Cambridge Massachusetts; ^4^ National Museum of Natural History, Smithsonian Institution Washington District of Columbia; ^5^ Coastal Research Center, Marine Science Institute University of California Santa Barbara Santa Barbara California

**Keywords:** *Chromis*, diet analysis, *Dascyllus*, foodwebs, niche theory, *Pocillopora*, predator prey interactions, species interactions

## Abstract

Theories involving niche diversification to explain high levels of tropical diversity propose that species are more likely to co‐occur if they partition at least one dimension of their ecological niche space. Yet, numerous species appear to have widely overlapping niches based upon broad categorizations of resource use or functional traits. In particular, the extent to which food partitioning contributes to species coexistence in hyperdiverse tropical ecosystems remains unresolved. Here, we use a molecular approach to investigate inter‐ and intraspecific dietary partitioning between two species of damselfish (*Dascyllus flavicaudus*, *Chromis viridis*) that commonly co‐occur in branching corals. Species‐level identification of their diverse zooplankton prey revealed significant differences in diet composition between species despite their seemingly similar feeding strategies. *Dascyllus* exhibited a more diverse diet than* Chromis*, whereas *Chromis* tended to select larger prey items. A large calanoid copepod, *Labidocera* sp., found in low density and higher in the water column during the day, explained more than 19% of the variation in dietary composition between *Dascyllus* and* Chromis*. *Dascyllus* did not significantly shift its diet in the presence of *Chromis*, which suggests intrinsic differences in feeding behaviour. Finally, prey composition significantly shifted during the ontogeny of both fish species. Our findings show that levels of dietary specialization among coral reef associated species have likely been underestimated, and they underscore the importance of characterizing trophic webs in tropical ecosystems at higher levels of taxonomic resolution. They also suggest that niche redundancy may not be as common as previously thought.

## 1 INTRODUCTION

Understanding the mechanisms that contribute to the maintenance of tropical biodiversity is central to predicting and maintaining the persistence of species‐rich assemblages as well as the ecological functions they provide. According to classic niche theory, species that exploit a similar range of resources are less likely to be able to coexist over long timescales than species that use different sets of resources (Chesson, 2000a; Hutchinson, 1961). Interspecific competition is thought to be reduced when species differ sufficiently along at least one dimension of their niche space, e.g., they may occupy different microhabitats (spatial niche partitioning), have asynchronous activity patterns (temporal niche partitioning) or consume different foods (dietary niche partitioning). However, many co‐occurring species show little or no apparent evidence of resource segregation (Sale, 1978; Siepielski & McPeek, 2010). This raises the question of whether the apparent niche overlap observed among many species pairs is a result of overly broad characterizations of resources (Kartzinel et al., 2015).

On coral reefs, arguably the most diverse marine ecosystem, there is a wealth of evidence for the existence of interspecific competition (Bonin, Boström‐Einarsson, Munday, & Jones, 2015). Fine‐scale spatial partitioning consistent with a niche based model is frequently observed (Actinopterygii: Robertson & Lassig, 1980; Gastropoda: Kohn, 1980; Malacostraca: Iglesias & Raso, 1999; Echinoidea: McClanahan, 1988; symbiotic dinoflagellate zooxanthellae: Sampayo, Franceschinis, Hoegh‐Guldberg, & Dove, 2007) and has been linked to competition for limited habitat availability (Munday, Jones, & Caley, 2001). Diurnal versus nocturnal activity patterns suggesting temporal partitioning also are conspicuous (Collette & Talbot, 1972). By contrast, the extent to which food partitioning contributes to species coexistence remains unresolved and subject to debate. The reconstruction of food webs to sufficient taxonomic levels has been challenging because of the high species richness involved in trophic interactions and the complex three‐dimensional structure in which these interactions occur (Enochs & Glynn, 2017). The vast majority of studies using morphological classification of semi‐digested food remains in gut contents concluded that coral reef associated taxa within feeding guilds (e.g., browsing herbivores, invertivores, or planktivores) had widely overlapping diets (Anderson et al., 1981; Bouchonnavaro, 1986; Depczynski & Bellwood, 2003; Gladfelter & Johnson, 1983; Harmelin‐Vivien, 1979; Hiatt & Strasburg, 1960; Hobson, 1974; Kulbicki et al., 2005; Longenecker, 2007; Pereira, Barros, Zemoi, & Ferreira, 2015; Randall, 1967; Ross, 1986; Talbot, Russell, & Anderson, 1978). Studies employing alternative strategies such as field observations of feeding behaviours (i.e., for corallivorous and herbivorous fish [Adam, Kelley, Ruttenberg, & Burkepile, 2015; Allgeier, Adam, & Burkepile, 2017; Pratchett, 2005, 2007; Pratchett & Berumen, 2008]), or combinations of gut content and stable isotope analyses (Ho et al., 2007; Nagelkerken, van der Velde, Wartenbergh, Nugues, & Pratchett, 2009) have identified more pronounced dietary differences between co‐occurring species. More recently, DNA‐based identifications of gut contents suggested very complex feeding behaviours previously obscured in studies that grouped food items into functional groups (Côté, Green, Morris, Akins, & Steinke, 2013; Leray, Agudelo, Mills, & Meyer, 2013; Leray, Boehm, Mills, & Meyer, 2012). For example, dietary comparisons among three coral‐dwelling invertivorous fish species using high‐throughput sequencing revealed that only ~20% of prey species had been consumed by more than one of the predator species (Leray, Meyer, & Mills, 2015). Despite providing preliminary insights, these studies included limited numbers of samples, and did not compare levels of intra‐ and interspecific differences in diet.

A diverse array of fishes and invertebrates associated with coral reefs capture and ingest planktonic animals from the water column as a feeding strategy. Planktivorous species are found in a majority of families of coral reef fishes (e.g., Pomacentridae, Holocentridae, Apogonidae, Balistidae) and many corals rely on heterotrophy in addition to autotrophy for nutrient acquisition (Houlbrèque & Ferrier‐Pagès, 2009). Numerous invertebrates living in holes and crevices created by the three‐dimensional structure of reefs also filter plankton, i.e., sponges, tunicates, bivalves, brittle stars, and polychaetes. Planktivory can be a dominant trophic mode, with up to 50% of total fish biomass comprised of planktivores (DeMartini, Friedlander, Sandin, & Sala, 2008), and it is a critical link in the transfer of organic matter from the water column to the benthic components of oligotrophic coral reef food webs (Hanson, Schnarr, & Leichter, 2016). Despite their abundance, diversity and functional role, the degree to which planktivorous organisms partition available food resources on coral reefs currently is not well quantified (see Frédérich, Fabri, Lepoint, Vandewalle, & Parmentier, 2009).

Pocilloporid branching corals provide habitat for a range of fishes and invertebrates, some of which share a planktivorous feeding strategy and thus potentially compete for food resources as they co‐occur among the branches of their coral host. The yellowtail dascyllus (*D. flavicaudus*) and blue green damselfish (*Chromis viridis*) reside among the branches of *Pocillopora* which afford a refuge from predation (Holbrook & Schmitt, 2002; Schmitt & Holbrook, 1999). While feeding, they swim in the water column directly above the corals to capture zooplankton. In exchange, they promote growth of their host by releasing nutrients in the form of nitrogenous waste (Holbrook, Brooks, Schmitt, & Stewart, 2008; Holbrook, Schmitt, & Brooks, 2011; Shantz, Ladd, Schrack, & Burkepile, 2015). Notably, they are commonly found together in branching corals feeding simultaneously during the day (Hanson et al., 2016; Holbrook et al., 2008, 2011). In this study, we hypothesize that given the lack of significant temporal and spatial niche partitioning, these two planktonic‐feeding fish are able to coexist, in part, because they partition their diet. *Chromis viridis* and *D. flavicaudus* have been considered to have broadly overlapping diets based on their highly similar feeding strategies and apparatus. Both rely on vision to detect and suction very small prey items in the water column and have only minor differences in the morphology of the jaw (Frédérich, Parmentier, & Vandewalle, 2006). *Dascyllus flavicaudus* has larger caniniform teeth and a stronger coronoid process than *C. viridis*.

A high‐throughput sequencing approach targeting the hypervariable mitochondrial Cytochrome c. Oxidase subunit I gene (COI) allowed us to characterize intra‐ and interspecific dietary differences between the two coral‐dwelling fishes with an unprecedented level of taxonomic resolution. We used the same sequencing approach on polyp tissues of the coral hosts to gain insights into the poorly known diet of a sessile and more passive consumer. To characterize the pool of prey that the three planktivorous species had access to and gain further insights into their feeding behaviour (i.e., feeding selectivity), we also conducted counts of zooplankton in surrounding waters.

The study was conducted in Moorea, French Polynesia, where an extensive library of COI DNA barcodes has been built by the BIOCODE project for >3,500 marine species (Meyer, 2017). This curated library of reference sequences was used to classify many sequences recovered from fish guts and coral tissue to the species level. Our results highlight the potential of the metabarcoding approach to decipher complex mechanisms of resource use in diverse ecosystems and across a wide range of consumers.

## 2 MATERIALS AND METHODS

### 2.1 Sample collection

In the lagoon of Moorea, French Polynesia, *Dascyllus flavicaudus* and *Chromis viridis* (hereafter referred to as *Dascyllus* and *Chromis*) are the two most abundant fish species occupying the branching structure of *Pocillopora* corals. Individuals of these two species are found residing in and hovering over coral colonies in large mono‐ or multi‐specific aggregations, and both species are visual planktivores that actively feed in the water column above their host corals during the day (Hanson et al., 2016; Holbrook et al., 2008, 2011). All fishes (including *Dascyllus* and *Chromis* individuals) resident on three experimental *Pocillopora eydouxi* colonies that had been outplanted to a sand flat in the Maharepa lagoon (17.4751°S, 149.8088°W) approximately two months prior to initiation of the study, were collected using small hand nets and the anaesthetic quinaldine (2‐methylquinoline) sulphate. Experimental corals, hereafter *Pocillopora* A, B and C, were representative of naturally occurring *P. eydouxi* colonies found within the Maharepa lagoon on the northern side of the island. Each of the three experimental colonies used in the study were approximately 1.0 m in diameter and 0.4 m in height, and were located at a depth of 2 m. The corals were spaced 10 m apart on a sand plain, which prevented target fish from moving between colonies (Schmitt & Holbrook, 1996, 1999). Fishes were collected after peak feeding as determined by visual observations made over several days (Brooks, personal communication) on 21 August 2008 between 1130 and 1200 hr. Fish were individually bagged underwater, placed on ice on the boat and frozen at –80°C immediately upon return to the laboratory. Three branch tips per experimental coral were clipped off and stored in sterile DMSO buffer (0.25 M EDTA [pH 7.5], DMSO, NaCl‐saturated) on the boat.

Zooplankton samples were collected during 60 min periods at night between 0200 hr and 0330 hr and again during the day between 1400 and 1530 hr over three days, August 18–20 2008, using submersible plankton pumps outfitted with precalibrated, internal flow metres, 200 μm mesh cod ends, and 2.5 cm inside diameter intake pipe openings (Alldredge & King, 2009). Pumps were deployed in pairs adjacent to each of the three outplanted *Pocillopora* colonies with one pump sampling the water column 0.3 m (mean ± *SD *= 0.32 ± 0.04 m) above the bottom and the other pump sampling 1.4 m (mean ± *SD *= 1.42 ± 0.09 m) above the bottom. Sampling heights corresponded to the maximum height of the outplanted corals above the bottom and the estimated maximum distance above the bottom where *Dascyllus* and *Chromis* had been observed to feed (Brooks, personal communication). Daytime plankton abundances in Moorea remain stable between noon when the fish were sampled and 1400–1530 hr when the plankton were sampled (Alldredge & King, 2009). Plankton samples were returned to the laboratory and preserved in 2% buffered formaldehyde for sorting, identification and enumeration using a dissecting microscope and plankton wheel. Flow meter data were utilized to standardize raw count data to numbers observed per cubic meter of water filtered. In four of the 36 samples, one to five *Dascyllus* or *Chromis* had entered the intake pipe and were captured in the cod end. These four samples were removed from the analyses because low plankton counts indicated substantial feeding in the cod end by the captured fish. Night samples were all collected while a bright three quarter moon was located mid‐sky. Height and day/night differences in abundance were tested for statistical significance using Student's *t*‐tests with the data log transformed where necessary to meet assumptions of normality.

Research was completed under permits issued by the Government of French Polynesia (Délégation à la Recherche) and the Haut‐commissariat de la République en Polynésie Francaise (DTRT) (Protocole d'Accueil 2005–2016).

### 2.2 Sample processing

Fishes were thawed on 2 September 2008, measured (total length in mm) and weighed (g wet weight). The entire digestive tract of each fish was then removed and individually stored at –20**°**C in numbered Eppendorf tubes containing 95% ethanol. Visual examination of the stomach contents of *Dascyllus* (*n* = 34) and *Chromis* (*n* = 27) revealed a range of undigested soft‐ (e.g., eggs) and hard‐bodied (e.g., copepods) prey from the morning's food intake. By contrast, the lower digestive tracts contained only undigested hard parts, most likely ingested the previous day. To obtain a snapshot of the diet for comparative analysis and avoid the misrepresentation of soft‐bodied taxa, we focused our molecular analysis on prey removed from the stomach only.

Using sterile tools, the contents of each fish stomach and coral tissues were transferred to individual 2 ml tubes for DNA extraction using the QIAGEN DNeasy Blood & Tissue kit following manufacturer's instructions. A small tissue subsample (1 cm × 1 cm) per branch tip was excised from the *Pocillopora* skeleton and thoroughly rinsed with DNA‐free water to remove excess DMSO buffer that could interfere with downstream analysis. The initial volume of lysis buffer was adjusted based on the amount of starting material if necessary, and samples were incubated with proteinase K overnight or until tissues were completely lysed. Genomic DNA was subsequently purified using the PowerClean DNA clean‐up kit (MO‐BIO) to minimize potential inhibition during Polymerase Chain Amplification (PCR). A negative control extraction and negative DNA purification were performed to check for potential contaminants.

### 2.3 High throughput sequencing

To reduce the per sample cost of sequencing on the Roche FLX platform, a hierarchical tagging approach, by which each sample is tagged with a unique combination of two indices, was implemented for sample multiplexing (Table S1). The first index was incorporated during PCR amplification using indexed PCR primers. The second tag was incorporated using a ligation of indexed adaptors (Leray, Haenel, & Bourlat, 2016; Leray, Yang, et al., 2013).

First, cleaned DNA extracts (*n* = 64) were used to amplify a hypervariable fragment (~313 bp) of the Cytochrome c. Oxidase subunit I (COI) region with versatile PCR primers mlCOIintF and jgHCO2198 (Geller, Meyer, Parker, & Hawk, 2013; Leray, Yang, et al., 2013) known to perform well across the diversity of marine invertebrates (Leray, Yang, et al., 2013). Despite some level of amplification bias, this primer set provides useful estimates of relative abundance as shown recently for benthic samples (Leray & Knowlton, 2015). A 6 bp index sequence was included at the 5’ end of each PCR primer (Tables S1 and S2), with each index sequence differing by at least 3 bp. These index sequences were shown to induce no significant bias in operational taxonomic units (OTU) detection (Leray & Knowlton, 2017). To avoid the erroneous assignment of reads due to tag jumping (Schnell, Bohmann, & Gilbert, 2015), we used identical indices on the forward and reverse primer for each individual sample (e.g., Sample 1: Index1‐mlCOIF/jgHCO‐index1; Sample 2: Index2‐mlCOIF/jgHCO‐index2). Because the co‐amplification of consumer DNA is known to prevent the recovery of some prey (Leray, Yang, et al., 2013; Vestheim & Jarman, 2008), consumer‐specific annealing blocking primers (Table S2) were included in each PCR reaction at 10 times the concentration of versatile primers. The PCR cocktail and touchdown temperature profile can be found in Leray, Yang, et al. (2013). Three PCR replicates were conducted per sample, pooled, gel excised to ensure complete removal of primer dimers, purified using QIAGEN MinElute columns and the product eluted in 12 μl of elution buffer. A PCR reaction performed with negative control extractions confirmed the absence of contaminants (no band on 1.5% agarose gel). PCR product concentration was normalized after quantification using the dsDNA Qubit Fluorometer (Invitrogen) and equimolar amounts of each sample were pooled, with each pool containing amplicons generated with each of the eight indexed primer pairs.

Second, a total of 500 ng of PCR product was used per pool for end‐repair and dA‐tailed using the NEBNext Quick DNA Sample Prep Reagent Set 2 chemistry (New England BioLabs) followed by the ligation of unique 454Multiplex Identifiers (Table S1) using the FLX Titanium Rapid Library MID Adaptors Kit (Roche). The ligated PCR products were purified using Agencourt AMPure beads (Beckman Coulter Genomics), eluted in 40 μl of TE buffer, and pooled prior to emulsion PCR and sequencing in two 454 runs. Details of the experimental design of each run are provided in Tables S1 and S2. Samples of both species were randomly assigned to each run. Note that additional samples unrelated to this study were also included in these runs.

### 2.4 Analysis of the sequence data

We used a data analysis procedure previously described in Leray, Yang, et al. (2013). First, flow files were generated from .sff files in Mothur and denoised using Pyronoise (Quince, Lanzen, Davenport, & Turnbaugh, 2011) implemented in Mothur (Schloss et al., 2009). Reads were then discarded if they (a) did not include forward and reverse primers and indices, (b) had more than one mismatch in primer index sequences, (c) had more than two mismatches in primer sequences, (d) had any ambiguous base calls (e.g., “N”), or (e) had any homopolymer regions longer than 8 bp. The fasta files were then demultiplexed based on primer indices in Mothur (Schloss et al., 2009), the sequences of both 454 runs were pooled and the data set was dereplicated using the trie function in QIIME (Caporaso et al., 2010). Following this initial quality filtering, the option “enrichAlignment” implemented in MACSE (Ranwez, Harispe, Delsuc, & Douzery, 2011) was used to align unique reads to the reference database of COI barcodes built by the Moorea BIOCODE (Leray et al., 2012; Meyer, 2017), an all‐taxa biodiversity inventory of the island ecosystem. MACSE performs alignments at the amino acid level and detects interruptions in the open reading frame due to nucleotide substitution or nucleotide insertion/deletion. We selected the invertebrate mitochondrial translation code and only retained sequences without any stop codons or frameshifts for subsequent analysis. To further reduce the variability in the data set and speed‐up the downstream clustering, we used an initial preclustering approach implemented in Mothur to merge reads differing by three or fewer bases. This algorithm first ranks sequences in order of their abundance and then merges rare sequences with more abundant sequences within the threshold specified (Huse, Welch, Morrison, & Sogin, 2010). Reads were then screened for chimeras using UCHIME (Edgar, Haas, Clemente, Quince, & Knight, 2011) implemented in Mothur before discarding all remaining preclusters represented by a single sequence (singletons).

The resulting quality filtered data set was used as an input for CROP (Hao, Jiang, & Chen, 2011), a Bayesian model that delineates OTUs based on the natural distribution of sequence dissimilarity. Rather than using a hard cut‐off (e.g., 5%), CROP generates clusters within user‐defined lower (‐l) and upper (‐u) bound levels of similarity to account for differences in rates of sequence evolution among taxonomic groups. We defined ‐l 3 and ‐u 4 because it was previously shown to delineate OTUs that closely reflect species grouping among marine invertebrates by providing the lowest frequency of false positives (splitting of single taxa) and false negatives (lumping of multiple taxa) (Leray, Yang, et al., 2013).

One representative sequence per OTU was used for taxonomic assignments following an iterative strategy. We ran similarity searches of each representative sequence against the Moorea BIOCODE reference database (BLASTn: word size = 11, e value = 1e‐20), GENBANK (BLASTn: word size = 11, e value = 1e‐20) and the Barcode of Life Data (search engine with default settings [Ratnasingham & Hebert, 2007]) to classify OTUs in three categories based on their level of similarity to a reference: >97%, 97%–85%, <85%. We considered that there was a species‐level “match” when the similarity to a reference sequence in one of the three databases was at least 97% (Machida, Hashiguchi, Nishida, & Nishida, 2009). In the case where an OTU matched multiple species at >97% similarity, it was assigned to the lowest common taxonomic rank. OTUs with a sequence similarity to a reference barcode between 97% and 85% were assigned to the phylum of the closest match as recommended by Ransome et al. (2017). Finally, OTUs with <85% similarity to a reference COI barcode were assigned to phyla using a phylogenetic approach implemented in the Statistical Assignment Package (SAP) (Munch, Boomsma, Huelsenbeck, Willerslev, & Nielsen, 2008). We allowed SAP to build 10,000 unrooted phylogenetic trees with 50 homologues retrieved from GENBANK (**>**70% sequence similarity) for each query sequence (i.e., each OTU representative sequence) and accepted taxonomic assignments at an 80% posterior probability cut‐off (Leray et al., 2015). OTUs that could not be confidently assigned using any of the methods above were labelled “unidentified”.

A sample by observation contingency table (later referred to as OTU table) summarizing the number of reads per OTU and per fish gut was built for downstream analysis.

### 2.5 Diversity analysis

To illustrate the extent of the sequencing effort, individual‐ and sample‐based rarefaction curves were built. The curves were computed by randomly resampling sequences and samples respectively at increasing levels of accumulation using EstimateS (Colwell, 2006). A curve that plateaus indicates a sufficient sampling effort as only rare OTUs remain to be detected.

Unequal numbers of reads can affect estimates of alpha and beta diversity because of the positive relationship between number of sequences and number of OTUs. Hence, a subsampling procedure (rarefaction) was used to create an alternative OTU table in which the number of reads of all samples was scaled down to the smallest number of sequences that a sample contained in the data set (259).

The rarefied OTU table was used to compute distance matrices of community dissimilarity based on the Jaccard and the Bray Curtis metrics within the R package Vegan (Oksanen et al., 2009). The input table was converted to a presence/absence matrix prior to calculating Jaccard (binary = true, in function metaMDS). Jaccard ranges from 0 to 1. A value of 0 indicates that samples have exactly the same OTU composition whereas a value of 1 indicates that samples do not have any OTU in common. Bray‐Curtis takes into account differences in abundance of reads between samples; a value of 0 indicates that samples are exactly identical in terms of OTU composition and abundance of reads whereas a value of 1 indicates that samples do not have any OTU in common. Bray‐Curtis gives less weight to rare OTUs than Jaccard, so that samples will have lower Bray‐Curtis values if they share abundant OTUs.

Patterns of species composition were visualized in two‐dimensional space using nonmetric multidimensional scaling (NMDS) plots. Differences in mean diet composition (position of groups of samples in multivariate space) were tested between species and between individual *Dascyllus* collected on different corals using PERMANOVA (Anderson, 2001). All statistical analyses were repeated with OTU tables rarefied down to 900 and 1,200 reads (which led to the removal of nine and 12 samples, respectively) to test for the robustness of ecological patterns to the loss of sequence data.

We further examined ontogenetic changes in fish diet by plotting pairwise Jaccard and Bray‐Curtis dissimilarities in relation to differences in size (total length in mm). Linear models with 95% confidence intervals were fitted to each plot. We used the statistical programming environment R Studio v. 0.98.1056 for the computation of all statistics, the “Vegan” package (Oksanen et al., 2009) for community analysis and the “ggplot2” package (Wickham, 2009) for graphics.

## 3 RESULTS

**Figure 1:**
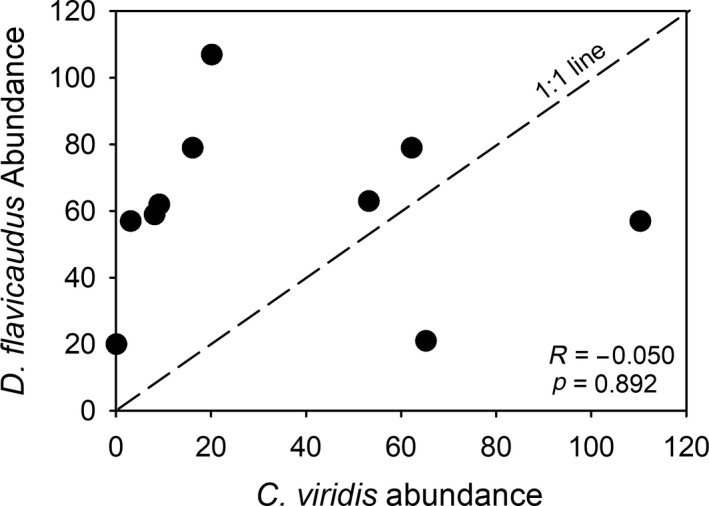
Co‐occurrence of planktivorous damselfishes surveyed on 10 *Pocillopora eydouxi* corals in the lagoon of Moorea

Surveys of damselfishes on 10 randomly chosen *P. eydouxi* corals in the Maharepa lagoon of Moorea revealed that 100% of adult *P. eydouxi* were occupied by at least one of the two species of damselfish (Figure 1) and 90% were occupied by both *Chromis* and *Dascyllus*. The abundances of *Chromis* and *Dascyllus* on these 10 colonies were not correlated (*F*_1,8 = _0.02, *p* = 0.8915; Figure 1). *Dascyllus* tended to be the more consistently abundant species. Several corals hosted large groups of both species (Figure 1).

A total of 42 *Dascyllus* and 32 *Chromis* were collected from the three experimental coral colonies. These abundances are characteristic of the numbers of these two species found on naturally occurring colonies of *P. eydouxi* (Holbrook et al., 2015) in the lagoons of Moorea. *Dascyllus* ranged in size from 13 to 84 mm TL (mean ± *SD *= 50 ± 16) and from 0.10 to 14.66 g wet weight (mean ± *SD *= 4.04 ± 3.45). *Chromis* ranged in size from 31 to 65 mm TL (mean ± *SD *= 51 ± 9) and from 0.49 to 4.34 g wet weight (mean ± *SD *= 2.48 ± 1.08). Both the mean length and weight of *Dascyllus* differed among the three coral colonies with individuals being significantly longer (*F*_2,39_ = 11.33, *p* = 0.0001) on *Pocillopora* B than on *Pocillopora* A and significantly heavier (*F*_2,39_ = 8.67, *p* = 0.0008) on *Pocillopora* B than on either *Pocillopora* A or C. Of these individuals, 34 *Dascyllus* (13 from *Pocillopora* A, 18 from *Pocillopora* B and three from *Pocillopora* C) and 27 *Chromis* (all from *Pocillopora* C) were used in the sequencing analysis.

### 3.1 Diversity and abundance of dietary items in fish stomachs

There were a total of 354,753 reads after denoising the data set but only 251,933 (71%) met our requirements as described in the Methods section. An additional 53,932 reads were discarded because they had interruptions in the open reading frame. Finally, Uchime detected 6,120 potentially chimeric reads that were also removed. The final data set contained 191,881 high quality reads (54%) with a number of reads per sample ranging from 279 to 8,003 (Overall: mean ± *SD *= 2,748 ± 690; *Dascyllus*: mean ± *SD *= 3,589 ± 1,380; *Chromis*: mean ± *SD *= 1,181 ± 472; *Pocillopora*: mean ± *SD *= 7,330 ± 660).

**Figure 2:**
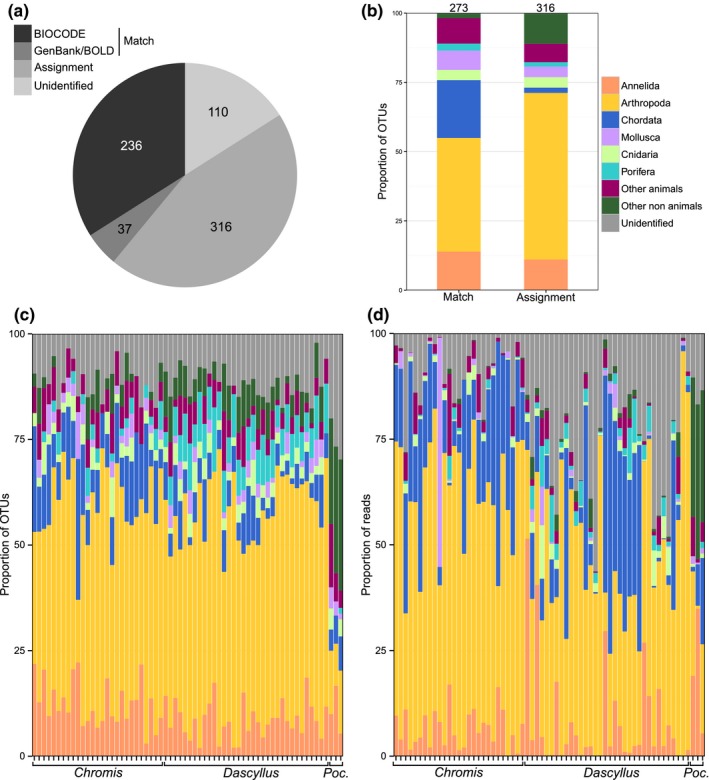
Diversity of operational taxonomic units (OTUs) recovered from dietary analysis of *Chromis viridis* (*n* = 27), *Dascyllus flavicaudus* (*n* = 34) and *Pocillopora eydouxi* (*n* = 3) using metabarcoding sequencing of COI. The proportion of OTUs identified (a) and their taxonomic breakdown (b) are illustrated. OTU diversity (c) and abundance (d) are also presented per individual fish and coral. An OTU was considered to match a reference sequence in the Moorea BIOCODE database, GenBank or BOLD if the level of similarity was higher than 97%. OTUs with < 97% similarity to reference COI barcodes were assigned to phyla using a Bayesian phylogenetic approach implemented in the Statistical Assignment Package (SAP) and closest Blast hits (if > 85% similarity, see Methods section). OTUs that could not be confidently assigned using any of the methods above were labelled “unidentified”. The category “Other animals” comprises Bryozoa, Chaetognatha, Echinodermata, Hemichordata, Nemertea, Platyhelminthes, Sipuncula and Xenacoelomorpha. The category “Other nonanimals” comprises members of Phaeophyceae, Rhodophyta, Dinophyceae, Bacillariophyta, Heterokonta, Amoebozoa and Fungi [Colour figure can be viewed at http://wileyonlinelibrary.com]

The Bayesian clustering tool CROP delineated a total of 716 OTUs. Among them, seven OTUs (189 reads) were identified as prokaryotes, seven OTUs (1,135 reads) as contaminants (e.g., *Homo sapiens*) and three OTUs (14,643 reads) as belonging to the host species (i.e., *D. flavicaudus*, *Chromis viridis* and* P. eydouxi*). These were removed from the data set, leaving a total of 699 eukaryotic OTUs (175,914 reads). A total of 236 OTUs matched a reference sequence in BIOCODE (>97% similarity) (Figure 2a) and 37 additional OTUs matched barcodes in GENBANK or BOLD (>97% similarity). Out of the 426 OTUs that did not match any reference barcode (<97% similarity), 316 could be assigned to a higher taxonomic level (>85% similarity in GenBank or SAP assignment, see Figure 2b), while 110 (15.8%) remained unidentified (labelled “unidentified” in Figure 2a).

OTUs spanned a total of 14 animal phyla, among which Arthropoda was the most diverse (302 OTUs), followed by Annelida (73 OTUs), Chordata (63 OTUs) and Mollusca (31 OTUs). Arthropoda and Chordata also comprised the highest number of sequences (45% and 21% respectively) followed by unidentified OTUs (16%). A majority of OTUs belonging to Chordata (90.4%), Bryozoa (87.5%) and Echinodermata (77.8%) matched representative barcodes in BIOCODE, GENBANK or BOLD (Figure 2b). In contrast, OTUs in the phyla Annelida, Arthropoda, Mollusca and Nemertea were less represented in barcode databases (52%, 37.1%, 61.3% and 11.1% matched, respectively).

A total of 36 OTUs (5%) were assigned to taxonomic groups known to have a strictly planktonic life cycle, whereas 201 OTUs (29%) belonged to taxa with a bipartite life cycle composed of a planktonic larval phase and a benthic adult phase. Planktonic taxa accounted for 11.8% and 44.9% of the sequences in the guts of *Dascyllus* (mean ± *SD *= 9 ± 8.1) and *Chromis* (mean ± *SD *= 41.6 ± 19.7), respectively, versus 4.1% of the sequences from *Pocillopora* tissues (mean ± *SD *= 3.8 ± 6). Five OTUs belonged to taxa known to be parasitic. The data set also contained 10 OTUs (236 sequences) belonging to major groups of protists, eight fungi OTUs (115 sequences) and representatives of major groups of multicellular algae (Phaeophyceae: five OTUs and 30 sequences; Rhodophyta: 17 OTUs and 2,727 sequences). Nonanimal OTUs were largely under‐represented in barcode libraries (10%, 25% and 9% of protists, fungi and algae OTUs had >97% match to reference barcodes, respectively).

Few OTUs were represented by a large number of sequences while many OTUs were rare (Figure S1). In total, 12.7% of OTUs were represented by two sequences only and 38.7% of OTUs contained <10 sequences. The phylum Mollusca comprised the highest proportion of rare OTUs (23% of doubletons) whereas Chordata and Porifera were mostly represented by OTUs with >10 sequences (73% and 92%, respectively) (Figure S2). As is commonly observed in metabarcoding analysis (Al‐Rshaidat et al., 2016; Leray & Knowlton, 2015; Leray et al., 2015), the most abundant OTUs were more likely to match reference barcodes with >97% similarity (32.6% of OTUs with two sequences; 42% of OTUs with at least 10 sequences) (Figure S3).

**Figure 3:**
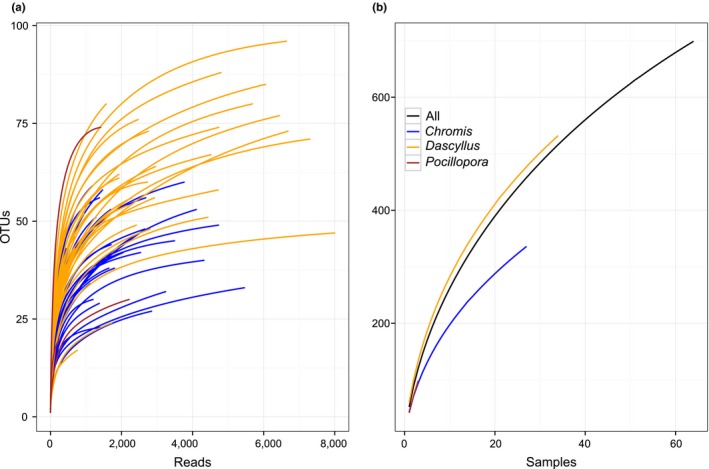
Individual (a) and sample (b) based rarefaction analyses. Rarefaction curves, built by randomly resampling sequences and samples respectively at increasing levels of accumulation, indicate whether the sequencing depth used in this study was sufficient to characterize the diversity of dietary items [Colour figure can be viewed at http://wileyonlinelibrary.com]

Individual‐based rarefaction curves reached a plateau between 1,000 and 2,000 reads for all samples indicating sufficient sequencing effort (Figure 3a). In other words, most of the OTUs in any particular gut were detected. By contrast, because of differences among individuals of each species, OTU numbers continued to climb with additional samples analysed. Thus examining the diet of additional fish and coral samples would have been required to better characterize the diversity of dietary items consumed on Moorea coral reefs at the time of sampling (Figure 3b). Both species of fish consumed a very diverse diet. The number of OTUs per sample ranged from 17 to 96 (Overall: mean ± *SD *= 52 ± 17; *Dascyllus*: mean ± *SD *= 60 ± 17; *Chromis*: mean ± *SD *= 43 ± 10; *Pocillopora*: mean ± *SD *= 41 ± 29) and was positively correlated with the number of reads per sample (Pearson: *r *= 0.47, *p* < 0.001) (Figure S4). The mean total number of OTUs was significantly different among species based on both the nonrarefied data set (ANOVA: *F*_2,61_ **= 9.5, *p* < 0.001) and the data set rarefied to 259 reads to account for differences in sequencing depth (ANOVA: *F*_2,61_ **= 6.3, *p* = 0.003). Post hoc Tukey tests indicated a higher mean diversity of OTUs in *Dascyllus* compared to *Chromis* (nonrarefied: *p* < 0.001; rarefied: *p* = 0.002) but no difference in the richness in the diet between *Pocillopora* and *Dascyllus* (nonrarefied: *p* = 0.12; rarefied: *p* = 0.75) or between *Pocillopora* and *Chromis* (nonrarefied: *p* = 0.97; rarefied: *p* = 0.71).

### 3.2 Patterns of dissimilarity in diet composition

**Figure 4:**
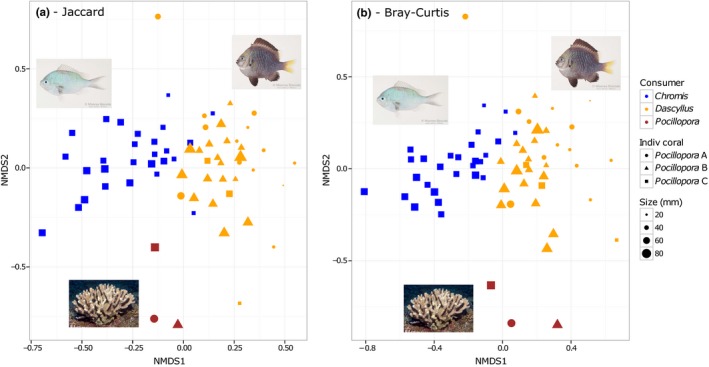
Ordination plots comparing the composition of prey items. Nonmetric multidimensional scaling plots were computed using Jaccard (stress = 0.22), an incidence‐based metric (a) and Bray‐Curtis (stress = 0.22), an abundance‐based metric (b). Note that the scale bar for size does not apply to *Pocillopora* samples. Beta diversity was calculated from the rarefied OTU table (*n* = 259) to control for differences in sequencing depth. Photos credit: Jeffrey Williams (fish), Gustav Paulay (coral) [Colour figure can be viewed at http://wileyonlinelibrary.com]

Using the data set rarefied to 259 reads to control for differences in sequencing effort, there was evidence for interspecific dietary partitioning. Mean pairwise β diversity values were higher between species than within species for both the Jaccard and Bray‐Curtis indices (Table 1). Samples of the three species were well separated on NMDS plots (Jaccard, Figure 4a; Bray‐Curtis, Figure 4b), and these differences in mean diet composition were statistically supported by PERMANOVA tests (Jaccard: *F* ^π^_2,61_ = 3.5, *R* ^2 ^= 0.1, *p* < 0.001; Bray–Curtis: *F* ^π^_2,61_ = 8.2, *R* ^2^ **= 0.21, *p* < 0.001) (Table 2). Differences in OTU composition were also significant between all pairs of species based on Jaccard (*Chromis* vs. *Dascyllus*: *F* ^π^_1,59_ = 4.7, *R* ^2^ **= 0.07, *p* < 0.001; *Chromis* vs. *Pocillopora*: *F* ^π^_1,28_ = 2.4, *R* ^2^ **= 0.08, *p* < 0.001; *Dascyllus* vs. *Pocillopora*: *F* ^π^_1,35_ = 2.2, *R* ^2^ **= 0.06, *p* < 0.001) and Bray–Curtis (*Chromis* vs. *Dascyllus*: *F* ^π^_1,59_ = 13.7, *R* ^2^ **= 0.19, *p* < 0.001; *Chromis* vs. *Pocillopora*: *F* ^π^_1,28_ = 4.2, *R* ^2^ **= 0.13, *p* = 0.002; *Dascyllus* vs. *Pocillopora*: *F* ^π^_1,35_ = 2.8, *R* ^2^ **= 0.07, *p* < 0.001).

**Table 1:** Intra‐ and interspecific dietary overlap measured using the Jaccard (below diagonal) and the Bray‐Curtis (above diagonal) dissimilarity metrics

Species	*Chromis*	*Dascyllus*	*Pocillopora*	Intraspecific means
*Chromis*		0.88	0.94	0.65
*Dascyllus*	0.86		0.96	0.79
*Pocillopora*	0.96	0.92		0.91
Intraspecific means	0.83	0.89	0.90	

Note

Beta diversity values were calculated using the data set rarefied to the lowest number of reads that a sample contained (259).

**Table 2:** Permutational multivariate analysis (PERMANOVA) testing inter‐ and intraspecific differences in diet

	Beta	Source of variation	*df*	SS	*F* Model	*r* ^2^	*p*‐value
Interspecific	Jaccard	Species	2	2.5	3.5	0.1	<0.001
		Residuals	61	21.8		0.9	
	Bray‐Curtis	Species	2	4.7	8.2	0.21	<0.001
		Residuals	61	17.4		0.79	
Intraspecific (*Dascyllus)*	Jaccard	Coral host	2	0.9	1.3	0.08	0.005
		Residuals	31	11		0.92	
	Bray‐Curtis	Coral host	2	0.7	1.2	0.07	0.14
		Residuals	31	9.8		0.93	

Note

Intraspecific diet partitioning was tested between individual *Dascyllus* collected on different coral hosts (A, B and C).

A total of 14 OTUs mostly belonging to Arthropoda and Chordata contributed 50% of the difference in Bray‐Curtis dissimilarity between *Chromis* and *Dascyllus* (Table 3). One OTU, identified as a very large planktonic copepod in the genus *Labidocera*, explained more than 19% of the variation in dietary composition between the two fish species. The same copepod also contributed 17.1% of the Bray‐Curtis dissimilarity between *Chromis* and *Pocillopora*. By contrast, no OTU contributed disproportionally to the dissimilarity between *Dascyllus* and *Pocillopora* (Table 3). One unidentified OTU, a red alga and an annelid explained 6.2%, 6.1% and 5.6% of the differences between these two species while >10 additional OTUs mostly belonging to Arthropoda and Annelida each contributed between 4% and 2% to the dissimilarity.

**Table 3:** Similarity Percentage Analysis (SIMPER) identifying the contribution of operational taxonomic units (OTUs) to differences between diets

Phylum	Lowest taxon	Contribution (%) to differences between species		
		*Chromis* versus *Dascyllus*	*Chromis* versus *Pocillopora*	*Dascyllus* versus *Pocillopora*
Arthropoda	*Labidocera* (Calanoid copepod)	**#19.3**	**#17.1**	*1.6
Unidentified		*5.6	**#2.7**	*6.2
Chordata	*Scarus psittacus* (Parrotfish)	*4.2	**#2.4**	*2.7
Arthropoda	*Farranula gibbula* (Cyclopoid copepod)	*2.9	#0.7	*2.8
Arthropoda	Maxillopoda	*2.6	#0.9	*2.2
Chordata	*Stegastes nigricans* (Pomacentrid)	**#2.5**	#2.3	*0.3
Unidentified		*2.0	#0.3	*1.9
Arthropoda	Chironomidae (Fly)	*1.7	#0.6	*1.5
Arthropoda		*1.7	#0.8	*1.7
Echinodermata	*Acanthaster planci* (Sea star)	**#1.6**	#1.5	*0.8
Arthropoda	*Scutellidium* (Harpacticoid copepod)	*1.6	#1.0	*0.8
Chordata	*Pseudocheilinus hexataenia* (Wrasse)	*1.6	#0.5	*1.1
Annelida	Polychaeta	**#1.5**	#1.5	x0.4
Chordata	*Cirripectes quagga* (Blenny)	**#1.4**	#1.4	0.0
Rhodophyta		*0.2	**x6.2**	**x6.1**
Annelida	Nereididae (Polychaete)	0.0	**x5.7**	**x5.6**
Rhodophyta		*0.1	**x4.0**	*3.8
Annelida	*Nematonereis unicornis* (Polychaete)	*0.2	**x3.7**	*3.6
Arthropoda		0.0	**x3.2**	**x3.2**
Unidentified		0.0	**x2.6**	**x2.5**
Rhodophyta		0.0	**x2.4**	**x2.4**
Chordata	*Labroides dimidiatus* (Labrid fish)	0.0	x2.4	**x2.3**
Chordata	*Thalassoma amplycephalum* (Labrid fish)	*1.1	#2.0	*2.3
Heterokonta		0.0	x2.2	**x2.2**

Note

OTUs that together contribute to 50% of the total difference in diet between each pairs of species are highlighted in bold (cumulated contribution of ranked OTUs). An OTU was considered to match a reference sequence in the Moorea BIOCODE database, GenBank or BOLD if the level of similarity was higher than 97%. OTUs with <97% similarity to reference COI barcodes were assigned to phyla using a Bayesian phylogenetic approach implemented in the Statistical Assignment Package (SAP) and closest Blast hits (if >85% similarity, see method section). OTUs that could not be confidently assigned using any of the methods above were labelled “unidentified”. The member of each pair that consumes more of the indicated prey item is marked with a symbol. #, *Chromis*; *, *Dascyllus*; x, *Pocillopora*.

The data set also revealed evidence for differences in OTU composition between *Dascyllus* from different *Pocillopora* coral hosts based on the Jaccard index (*F* ^π^_2,31_ = 1.3, *R* ^2^ **= 0.08, *p* = 0.005), whereas no significant differences were found using the Bray‐Curtis index (*F* ^π^_2,31_ **= 1.2, *R* ^2^ **= 0.07, *p* = 0.14) (Table 2). Pairwise tests showed that prey composition in stomachs of *Dascyllus* collected on *Pocillopora* C, where *Chromis* also occurred, was not significantly different from *Dascyllus* collected from *Pocillopora* A (Jaccard: *F* ^π^_1,14_ = 1.17, *R* ^2^ **= 0.08, *p* = 0.11; Bray–Curtis: *F* ^π^_1,14_ = 0.92, *R* ^2^ **= 0.06, *p* = 0.58) and *Pocillopora* B (Jaccard: F^π^_1,19_ = 1.16, *R* ^2^ **= 0.05, *p* = 0.14; Bray–Curtis: *F* ^π^_1,19_ = 0.94, *R* ^2^ **= 0.05, *p* = 0.55), where *Chromis* was absent. We repeated NMDS and PERMANOVA analyses with data sets rarefied down to 900 and 1,200 reads. Results show that the patterns are robust to the loss of sequence data (Table S3).

The number of reads reflects the individual size as well as the abundance of each OTU consumed, with larger or more numerous prey items generating more reads. Thus, the presence (or absence) of food items in the gut that could be clearly identified and assigned to a major taxonomic category was assessed to further elucidate differences in diet among the three species (Table 4) and better compare them to plankton availability. Almost all of the OTUs identifiable as calanoid and cyclopoid copepods were detected in the fish stomachs, but there were almost twice as many reads of the OTUs identifiable as harpacticoid copepods in samples of *Dascyllus* than *Chromis*. There were more OTUs identifiable as amphipods, isopods, ostracods, tanaids, gastropods, and polychaetes in stomach samples of *Dascyllus* than *Chromis* indicating that *Dascyllus* has a much broader diet than *Chromis*. Samples of *Dascyllus* had a greater diversity of fish OTUs, most certainly consumed as eggs. *Chromis* stomach samples yielded more reads of the OTUs identified as *Labidocera* sp. and larvae of shrimps and crabs compared to *Dascyllus* stomach samples. Only a few OTUs of cyclopoid and harpacticoid copepods, shrimp, polychaetes, appendicularians, and gastropods were present in *Pocillopora* tissues. Mysids, and the cephalochordate, *Branchiostoma* sp., were absent from the guts analysed.

**Table 4:** Summary of metazoan OTUs by taxonomic group

	Sum of Reads			Number of identifiable OTUs in gut			Total identifiable OTUs
	CV	DC	P	CV	DC	P	
*Labidocera* sp.	24,545	73	152	1	1	1	1
Other Calanoids	838	757	0	8	8	0	9
Cyclopoids	1,129	9,929	7	10	11	1	12
Harpacticoids	1,176	3,307	8	10	18	2	19
Amphipods	6	127	0	2	7	0	7
Gnathid Isopods	0	0	0	0	0	0	0
Other Isopods	6	138	0	1	4	0	5
Ostracods	1	134	0	1	3	0	3
Mysids	0	0	0	0	0	0	0
Tanaids	0	75	0	0	2	0	2
Crab (larvae)	119	494	0	8	6	0	12
Shrimp (larvae)	1,592	159	29	17	9	1	19
Stomatopod (larvae)	16	24	0	2	1	0	2
Gastropod (larvae)	219	936	33	8	12	2	15
Polychaetes	2,192	6,440	807	29	45	4	59
Appendicularia	0	4	0	0	1	0	1
*Branchiostoma* sp.	0	0	0	0	0	0	0
Fish	12,572	23,112	508	25	39	7	57

Note

Sum of Reads is the total number of reads found for each taxonomic group. The number of identifiable OTUs consumed by each predator and the total number of OTUs identifiable are indicated. Fish were mostly consumed as fish eggs. *Labidocera* sp. was singled out from other calanoid copepods. The complete OTU table can be downloaded from Figshare (https://doi.org/10.6084/m9.figshare.7551026).

### 3.3 Ontogenetic dietary shifts

**Figure 5:**
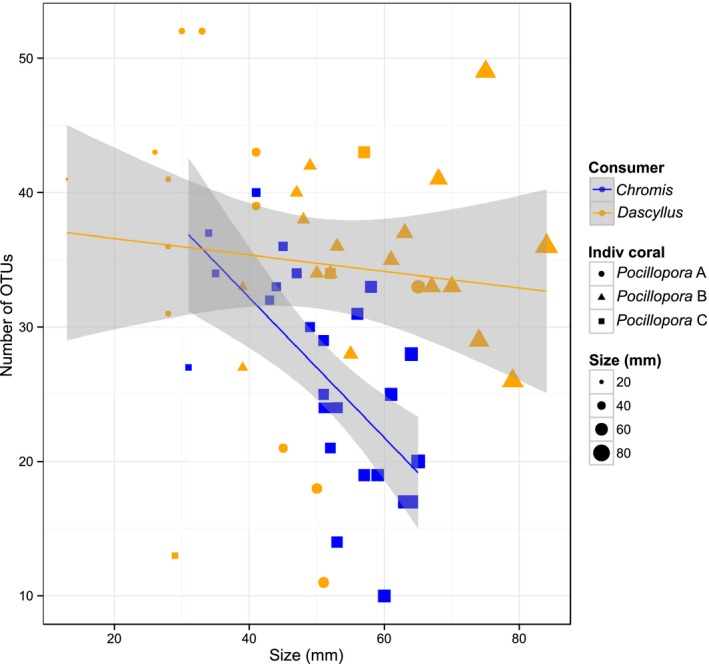
Diversity of OTUs in relation to fish size (total length in mm). A linear model is fitted with 95% confidence intervals. *Chromis*: *Y* = 53–0.5*X, adjusted *r* ^2^ = 0.39, *p* < 0.001; *Dascyllus*: *Y* = 37.8–0.1*X, adjusted *r* ^2^ = 0.01, *p* = 0.5. The number of OTUs was calculated from the rarefied data set (*n* = 259) to control for differences in sampling effort [Colour figure can be viewed at http://wileyonlinelibrary.com]

**Figure 6:**
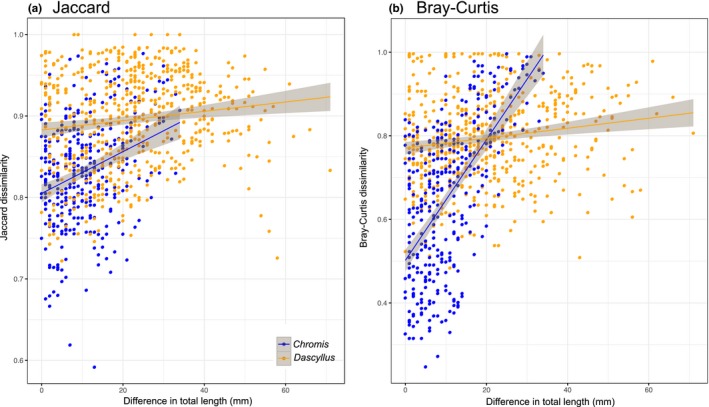
Correlation between prey composition and differences in fish size (total length in mm). Pairwise differences in prey composition were computed using (a) Jaccard, an incidence based metric and (b) Bray‐Curtis, an abundance based metric. Linear models with 95% confidence intervals were fitted to each plot. Jaccard, *Chromis*: *Y* = 0.80 + 0.002*X, adjusted *r* ^2^ = 0.09, *p* < 0.001; Jaccard, *Dascyllus*: *Y* = 0.88 + 0.001*X, adjusted *r* ^2^ = 0.02, *p* = 0.001; Bray‐Curtis, *Chromis*: *Y* = 0.50 + 0.014*X, adjusted *r* ^2^ **= 0.37, *p* < 0.001; Bray‐Curtis, *Dascyllus*: *Y *= 0.77 + 0.001*X, adjusted *r* ^2^ **= 0.02, *p* < 0.001. Beta diversity was calculated from the rarefied OTU table (*n* = 259) to control for differences in sequencing depth [Colour figure can be viewed at http://wileyonlinelibrary.com]

There was a significant linear negative relationship between fish size (total length) and the number of OTUs in the stomach contents of *Chromis* (adjusted *r* ^2^ **= 0.39, *p* < 0.001) but not for *Dascyllus* (adjusted *r* ^2^ **= 0.01, *p* = 0.5) (Figure 5). Prey composition appeared to shift during fish ontogeny based on NMDS plots (Figure 4a,b). The gradual change in diet was particularly marked for *Chromis* when prey abundance was taken into account in calculations of β diversity. A simple linear model explained 37% of the variance in the relationship between the Bray‐Curtis index and differences in fish size (total length) (Figure 6b). The relationship was also significant for *Dascyllus* (*p* < 0.001) but the model explained a much lower proportion of the variance (adjusted *r* ^2^ **= 0.02). When taking into account presence‐absence to calculate β diversity (Jaccard), the relationships also were significant for both species (*Chromis*: *p* < 0.001;* Dascyllus*: *p* < 0.001), but the model explained relatively low amounts of the variance (*Chromis*: adjusted *r* ^2^ **= 0.09;* Dascyllus*: adjusted *r* ^2^ **= 0.02) (Figure 6a).

### 3.4 Diversity and abundance of dietary items collected by plankton pumps

Zooplankton diversity in this hyperdiverse system was expected to be very high and dominated by crustacean larvae and copepods. Given the impossibility of identifying almost all larval forms to species morphologically and the difficulty of identifying most of the copepods to species, identification of the ambient zooplankton was generally at a higher taxonomic level than provided by the sequencing approach. Available monetary resources precluded sequencing the ambient zooplankton samples. However, these samples still provided important insights into the food availability for all three planktivores studied. Both the abundance and diversity of planktonic prey items were significantly higher at night at both depths (*t *= 6.573, *p *= <0.001) when *Chromis* and *Dascyllus* do not feed (but the *Pocillopora* does) compared to during the day when the fish and coral both feed. Zooplankton was relatively homogeneously distributed at the two depths during the day. Daytime abundances were low, averaging <20 items m^−3^ at both depths (Table 5). Daytime plankton samples were dominated by small copepods which made up 41% of the available prey items at 1.4 m and 31% at 0.3 m height above the bottom, followed by eggs which contributed 33% at 1.4 m and 51% at 0.3 m and gastropod protoconchs which contributed 13% at 1.4 m and 6% at 0.3 m (Table 5). Large‐bodied taxa such as amphipods, isopods, crab and shrimp larvae, other crustacean larvae, the predatory calanoid copepod *Labidocera* sp. and polychaetes were rare in the daytime samples but relatively abundant at night. Eggs were about half as abundant at night as during the day (Table 5).

**Table 5:** Abundance of major zooplankton taxa in the water column at two heights above the experimental corals

	Night				Day			
	1.4 m		0.3 m		1.4 m		0.3 m	
	No. m^−3^	%	No. m^−3^	%	No. m^−3^	%	No. m^−3^	%
*Labidocera* sp.	2.5 ± 2.2	3.0	3.0 ± 6.1	7.8	0.1 ± 0.2	0.7	0.1 ± 0.1	0.3
Other Calanoids	23.7 ± 12.1	28.6	12.5 ± 4.8	32.2	2.7 ± 2.2	14.9	1.1 ± 0.4	7.1
Cyclopoids/Harpacticoids	8.0 ± 2.0	9.7	9.1 ± 5.8	23.4	4.5 ± 2.0	24.9	3.7 ± 1.8	23.8
Amphipods	5.0 ± 3.0	6.0	1.4 ± 3.1	3.7	0.3 ± 0.3	1.7	0.3 ± 0.3	1.8
Gnathid Isopods	0.3 ± 0.3	0.3	3.4 ± 1.6	8.9	0.0 ± 0.1	0.2	0.6 ± 0.5	4.2
Other Isopods	0.9 ± 0.6	1.1	1.4 ± 1.8	3.5	0.1 ± 0.2	0.7	0.3 ± 0.3	2.0
Ostracods	0.9 ± 1.2	1.1	0.2 ± 0.3	0.5	0.4 ± 0.4	2.1	0.4 ± 0.3	2.4
Mysids	0.4 ± 0.4	0.5	0.1 ± 0.1	0.2	0.0 ± 0.0	0.0	0.0 ± 0.0	0.0
Tanaids	0.0 ± 0.0	0.0	<0.1 ± 0.0	0.0	0.0 ± 0.1	0.2	0.1 ± 0.1	0.7
Crab zoea	10.4 ± 7.1	12.5	0.2 ± 0.3	0.5	0.5 ± 0.3	2.6	<0.1 ± 0.0	0.0
Crab megalops	0.3 ± 0.4	0.4	0.1 ± 0.1	0.2	0.1 ± 0.1	0.4	0.1 ± 0.1	0.4
Other decapod larvae	16.9 ± 10.7	20.4	0.5 ± 0.3	1.3	0.7 ± 0.6	3.8	0.0 ± 0.0	0.0
Shrimp	0.2 ± 0.2	0.2	0.4 ± 0.2	1.1	0.0 ± 0.0	0.0	<0.1 ± 0.0	0.0
Stomatopod larvae	0.0 ± 0.0	0.0	0.0 ± 0.0	0.0	0.0 ± 0.0	0.0	0.0 ± 0.0	0.0
Gastropods	7.5 ± 6.0	9.0	1.4 ± 1.4	3.6	2.4 ± 1.5	13.2	0.9 ± 0.5	6.0
Polychaetes	3.8 ± 2.3	4.6	1.1 ± 0.8	2.8	0.2 ± 0.3	1.3	0.1 ± 0.3	0.7
Appendicularia	0.2 ± 0.4	0.3	0.1 ± 0.2	0.4	0.0 ± 0.1	0.2	<0.1 ± 0.0	0.0
*Branchiostoma* sp.	0.0 ± 0.0	0.0	<0.1 ± 0.0	0.0	0.0 ± 0.0	0.0	0.0 ± 0.0	0.0
*Spadella* sp.	<0.1 ± 0.0	0.0	0.0 ± 0.0	0.0	0.0 ± 0.0	0.0	0.0 ± 0.0	0.0
Fish larvae	0.1 ± 0.1	0.1	0.0 ± 0.0	0.0	0.0 ± 0.0	0.0	0.0 ± 0.0	0.0
Eggs	2.0 ± 0.7	2.4	3.8 ± 1.6	9.7	6.1 ± 3.6	33.2	7.8 ± 2.9	50.6
Total	83.0 ± 37.4	100.0	38.8 ± 9.1	100.0	18.3 ± 8.7	100.0	15.5 ± 2.0	100.0

## 4 DISCUSSION

Resolving whether species and individuals partition their food resources is key to understanding mechanisms potentially allowing a diverse community of species to coexist on coral reefs. The lack of taxonomic resolution in prey identification, the diversity of potential food resources, and the structural complexity of the reef habitat have hindered efforts to confidently identify and quantify dietary differences. It has been particularly challenging for planktivores that feed on small particulate prey, although they represent one of the dominant feeding guilds on coral reefs. In this study, we identify for the first time, pronounced interspecific dietary differences among co‐occurring planktivorous fish species, which suggest the role of dietary selectivity in promoting coexistence on coral reefs.

Our investigation focused on the molecular dietary analysis of a coral host and two of its associated planktivorous fish species using high throughput sequencing of a hypervariable mitochondrial gene. Although this approach has been used extensively in microbial ecology, it has seldom been applied to decipher food webs (Leal & Ferrier‐Pagès, 2016). It is now a powerful complement to traditional analyses of diets (i.e., morphological analysis of gut contents) owing to methodological improvements (i.e., primer sets for short amplicons) combined with the development of large curated databases of metazoan sequences (Machida, Leray, Ho, & Knowlton, 2017).

We found a diverse range of prey with 699 OTUs belonging to 14 phyla, of which 273 (39%) matched a reference barcode. This is relatively high for a hyperdiverse tropical ecosystem and probably reflects the extensive efforts of the BIOCODE project (Meyer, 2017) at cataloguing and barcoding the marine life of the island of Moorea. Species that remain uncollected likely belong to morphologically cryptic species, benthic taxa living at greater depths consumed as eggs or larvae, or micro‐zooplanktonic taxa still under‐represented in the database. Nonetheless, species level prey resolution helped identify 201 benthic prey species (29% of OTUs and 35.7% of the sequences) consumed at the larval or egg stages. Although OTUs with a strictly planktonic life cycle were not as diverse (36), they represented 23.4% of the total number of sequences in the data set with an average contribution of holoplankton 4.6 and 10.9 times higher in the diet of *Chromis* than in *Dascyllus* and *Pocillopora*, respectively. These estimates based on sequence data are slightly lower than previous estimates made using stable isotopes (34%–55% for *Dascyllus* in the lagoon of Moorea [Hanson, 2011]) possibly because some planktonic OTUs remained unidentified in our data set. Nevertheless, they confirmed that planktivores are an essential trophic link and that not all species contribute equally to the transfer of nutrients from the pelagic to the benthic food web. It is also possible that some of the rarer species catalogued as prey for the fish may in fact have been items from the guts of the prey items themselves as some of the zooplankton, including *Labidocera*, feed on other zooplankton.

Our findings reveal that despite having highly similar feeding behaviours and morphologies, the two planktivorous fish species that co‐occur in close proximity in branching corals consumed very different portions of the available resources. Levels of interspecific dietary dissimilarity were higher than levels of intraspecific dietary dissimilarity (Table 1), a pattern consistent with predictions of niche theory (Chesson, 2000b). Interestingly, *Dascyllus* did not significantly shift its diet in the presence of *Chromis*. This suggests that the two species have intrinsic differences in their feeding behavior and hence, might not strongly compete for food (i.e., no competitive displacement). *Chromis* and *Dascyllus* are both visual particulate feeders that use suction feeding to capture individual evasive prey items. However, the presence of stronger caniniform teeth and an enlarged coronoid process in the mandible give *Dascyllus* an ability to seize prey in addition to sucking prey (Frédérich et al., 2006), which could explain why they are able to feed on a wider array of resources. Although it has never been reported in the literature and we never observed this behaviour, the range of benthic prey in stomachs of *Dascyllus* could suggest that they also pick prey off the substrate as an alternative feeding strategy. Zooplanktivorous fish rely on their vision for feeding, and the dietary specialization of *Chromis* and *Dascyllus* may be driven largely by their ability to detect prey of different pigmentation, shape or behaviour. For example, the visibility of prey to a fish has been shown to depend upon how well it is able to discriminate various body structures from the background (i.e., light contrast) (Lazzaro, 1987), which is a function of the physical properties of the eyes. The successful capture of prey detected is also contingent on whether the fish is able to discriminate among individual moving prey with various motion patterns and escape strategies. For example, the predatory calanoid copepod *Labidocera* sp., that explained more than 19% of the variation in dietary composition between the two fish species (Table 3), has a characteristic gliding behaviour that might enable *Chromis* to detect it more readily, or might prevent *Dascyllus* from catching it.

Prey size may also play a role in dietary separation. The *Labidocera*, decapod larvae, and polychaetes primarily consumed by *Chromis* are among the largest prey available. Measurements of the carbon content of plankton from Moorea revealed that *Labidocera*, decapod larvae, crab megalops, and polychaetes averaged 42 μg C, 18 μg C, 70 μg C, and 44 μg C animal^−1^ respectively, while the small copepods, crustaceans and gastropod larvae favoured by *Dascyllus* averaged between 2 and 16 μg C animal^−1^ (Alldredge & King, 2009). At an average of 27 μg C/animal, amphipods favoured by *Dascyllus* were an exception although still smaller than most of the larger prey consumed by *Chromis*. We deliberately singled *Labidocera* sp. out from the other copepod taxa because it was so much larger (1.5–2.5 mm length) than all the other copepods taxa combined (0.4–1.2 mm length) (Alldredge & King, 2009) as well as being a highly significant food item. While prey mainly consumed by both fish species (i.e., *Labidocera* sp., decapod larvae, amphipods, isopods, and polychaetes) are more abundant in the water column at night, both night‐time video recordings and diver observations indicate that *Chromis* and *Dascyllus* shelter from predators in their host corals at night and do not feed during that period (Holbrook & Schmitt, 2002; Yahel, Yahel, Berman, Jaffe, & Genin, 2005; Brooks personal observation).

Alternatively, concentrations of certain prey species in the stomachs of *Chromis* and *Dascyllus* that exceed their measured concentrations in the water column could reflect either a high degree of selectivity on the part of the two damselfishes or nonrandom collection or underestimation of the available prey by the plankton pumps. However, while the plankton pumps tend to collect fewer zooplankton than diver‐towed nets, the diversity of the organisms caught is similar (Alldredge & King, 2009). Moreover, *Dascyllus* is known to selectively feed on several species of copepods including *Corycaeus* sp. (Hanson et al., 2016)*,* a genus sometimes seen as synonymous with the *Farranula* sp. on which it also selectively fed in our study. Dietary differences also may be caused by the uneven spatial distribution of prey. The schooling behaviour of both *Chromis* and *Dascyllus* allows exploration of larger volumes of water. In the absence of predators, the groups swim high in the water column. Several studies have reported marked vertical stratification of zooplankton on reefs, with some taxa consistently more abundant towards the surface (Heidelberg, O'Neil, Bythell, & Sebens, 2010; Holzman, Reidenbach, Monismith, Koseff, & Genin, 2005). Alldredge and King (2009) showed that copepod and veliger larvae were 3–8 times more abundant in the upper 50 cm of the shallow back reef of Moorea (2.4 m) during the day. This vertical zonation was driven by upward swimming in response to strong predation pressure near the sea floor. During this study, we only sampled for zooplankton near the bottom and in midwater, where, as expected, plankton abundance was low and relatively unstratified during the day (Alldredge & King, 2009). However, prey abundances would be expected to be higher nearer the surface. *Labidocera* in particular is highly attracted to light and was likely much more abundant nearer the surface during the day (Alldredge & King, 2009) suggesting that *Chromis* may occasionally forage higher up in the water column. Although we did not quantify patterns of foraging by the two species of fish in the water column during this study, larger individuals of both species tend to feed in the upper part of the water column while smaller individuals feed closer to their coral host (Brooks, personal communication). In addition, *Chromis* do tend to feed higher in the water column than *Dascyllus*, which indicates that vertical segregation is likely responsible for both inter‐ and intraspecific dietary differences in both species.

As expected, *Pocillopora* has a very distinct diet owing to its feeding mode and its position in the water column. Zooplankton are a significant source of nutrients for scleractinian corals, and are essential for the maintenance of metabolic processes and skeletal growth (Ferrier‐Pagès, Witting, Tambutté, & Sebens, 2003; Houlbrèque, Tambutté, & Ferrier‐Pagès, 2003; Palardy, Rodrigues, & Grottoli, 2008). *Pocillopora* uses two main feeding strategies to capture zooplankton during both day and night (Séré, Massé, Perissinotto, & Schleyer, 2010). First, polyps immobilize individual prey using nematocyst stings and pull them towards the mouth. Second, polyps extrude mucus webs that they then pull back into the mouth once several prey have been trapped. This passive feeding strategy likely broadens the range of prey ingested. Our study revealed that *Pocillopora* tissues contained a diversity of fish, present in the mucus as eggs, small larvae and possibly in the form of faeces. Coral tissue also contained numerous microalgae, confirming the importance of herbivory in coral nutrition (Leal et al., 2014).

The molecular approach used here provided dietary information with an unprecedented level of resolution. Yet, it also has shortcomings (Creer et al., 2016; Leray & Knowlton, 2016; Pompanon et al., 2012). For example, primer mismatches likely create biases in the relative number of sequences per taxa (Deagle, Thomas, Shaffer, Trites, & Jarman, 2013). Polymerase chain reaction may also co‐amplify secondary prey (i.e., the prey of a prey), thus artificially increasing prey richness (Sheppard et al., 2005) and inflating dietary partitioning. The detection of secondary prey is unavoidable with this sequencing approach. However, they likely make up negligible amounts of DNA, are likely highly digested in comparison to primary prey, and therefore likely account for very few sequences in the data set (Sheppard et al., 2005). For example, OTUs assigned to nonmetazoan groups (e.g., protists, fungi and algae) that are possible secondary prey for the planktivores account for only 1.8% of the sequences in this data set. Our analyses showed clear patterns of dietary partitioning regardless of the beta diversity metric used (i.e., equal or little weight given to rare OTUs) and sequencing effort (i.e., level of rarefaction), pointing to the value of these tools for understanding the complexity of trophic interactions and the role of dietary specialization for the maintenance of biodiversity on coral reefs.

## AUTHOR CONTRIBUTION

A.J.B., A.L.A., S.J.H., R.J.S., C.P.M. and M.L. designed the study. A.J.B. and A.L.A. collected the samples. M.L. conducted the laboratory work. M.L. and J.Y.Y. analyzed the molecular data. C.P.M. and N.K. contributed reagents. A.L.A. conducted the plankton survey. M.L. wrote the manuscript with input from A.L.A. and A.J.B. All authors discussed the results and contributed to the final version of the manuscript.

## DATA ACCESSIBILITY

Raw sequence data files are available from Figshare (https://doi.org/10.6084/m9.figshare.5808618 and https://doi.org/10.6084/m9.figshare.5808621) and the NCBI Short Read Archive (BioSample accessions: SAMN10780924‐SAMN10780987). Sequences of PCR primers and 454 multiplex identifier (https://doi.org/10.6084/m9.figshare.7550183), a description of the multiplexing strategy (https://doi.org/10.6084/m9.figshare.7550180) and the complete OTU table can also be downloaded from Figshare (https://doi.org/10.6084/m9.figshare.7551026).
