# Supplementary material for: Dietary partitioning promotes the coexistence of planktivorous species on coral reefs
Source: Mol Ecol. 2019 May 13;28(10):2694–710. doi: 10.1111/mec.15090 (PMC6852152; doi:10.1111/mec.15090)
Supplement: Supplementary file 1 [file MEC-28-2694-s001.pdf]

SUPPLEMENTARY MATERIALS FOR:

Dietary partitioning promotes the coexistence of planktivorous species on coral reefs

Matthieu Leray[1]*, Alice L. Alldredge[2,3], Joy Y. Yang[4], Christopher P. Meyer[5], Sally J. Holbrook[2,3], Russell J. Schmitt[2,3], Nancy Knowlton[5], Andrew J. Brooks[3]

[1] Smithsonian Tropical Research Institute, Smithsonian Institution, Panama City, Balboa, Ancon, Republic of Panama

[2] Department of Ecology, Evolution and Marine Biology, University of California Santa Barbara, Santa Barbara, CA, 93106, USA

[3] Coastal Research Center, Marine Science Institute, University of California Santa Barbara, Santa Barbara, CA, 93106, USA

[4] Computational and Systems Biology, Massachusetts Institute of Technology, Cambridge, MA, USA

[5] National Museum of Natural History, Smithsonian Institution, Washington, DC 20013, USA

*corresponding author, E-mail: leray.upmc@gmail.com

Figure S1 Rank abundance distribution of OTUs

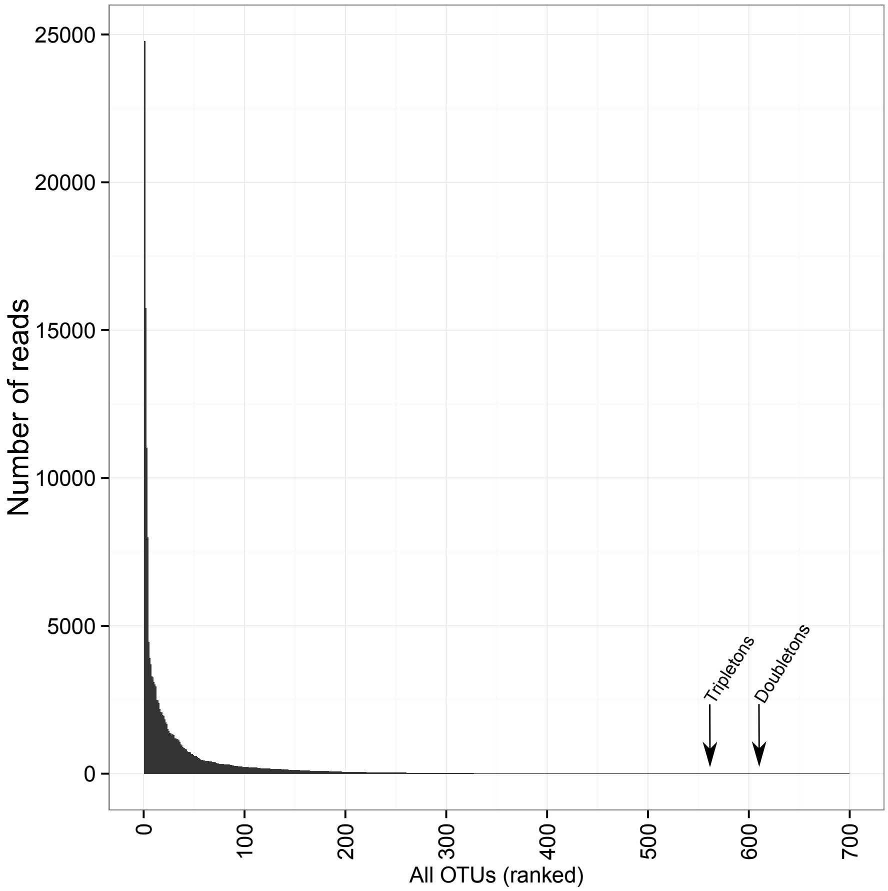

Figure S2 Abundance distribution of OTUs in major taxonomic groups. The category "Other animals" comprises Bryozoa, Chaetognatha, Echinodermata, Hemichordata, Nemertea, Platyhelminthes, Sipuncula and Xenacoelomorpha. The category "Other non animals" comprises members of Phaeophyceae, Rhodophyta, Dinophyceae, Bacillariophyta, Heterokonta, Amoebozoa and Fungi.

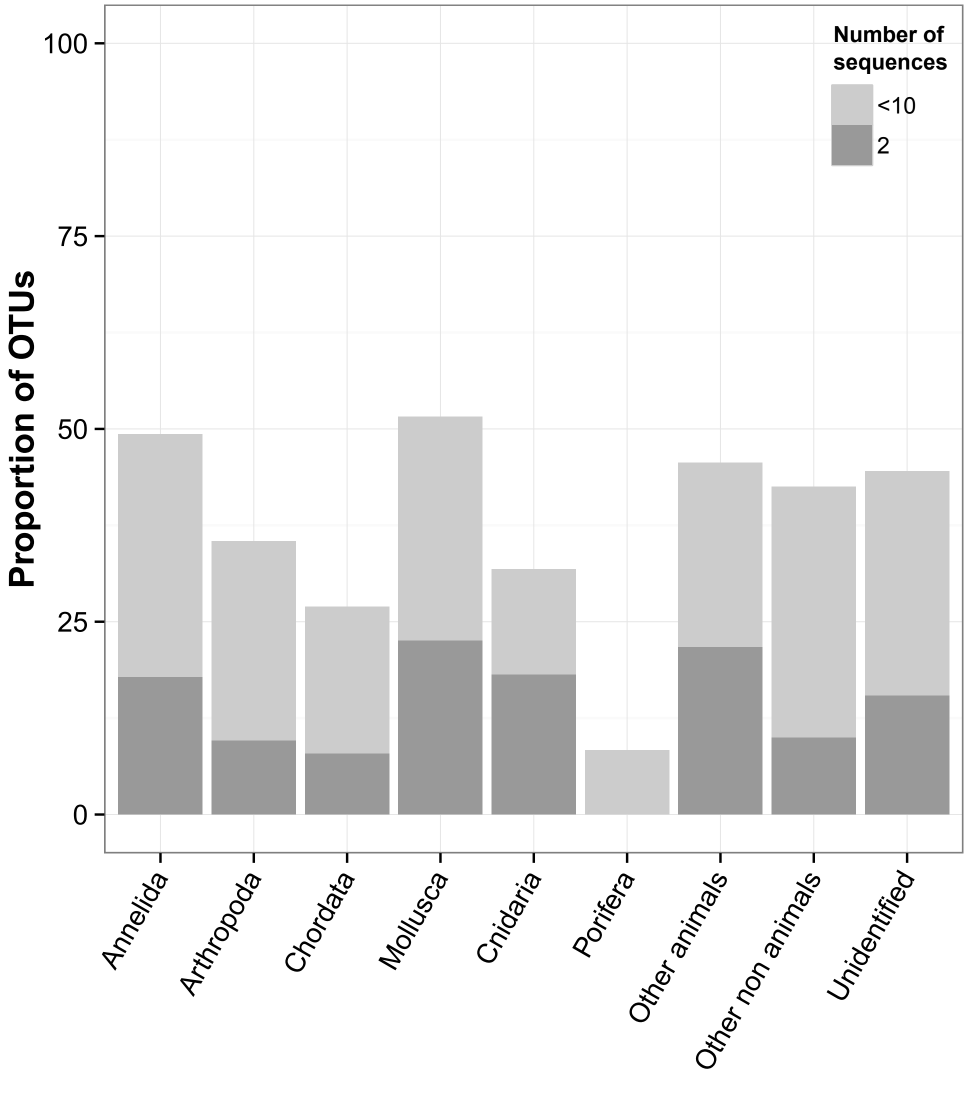

Figure S3 OTU identification according to the number of sequences. >97% match: match to a reference sequence in the Moorea BIOCODE database, GenBank or BOLD with a level of similarity higher than 97%. Assigned: OTUs with <97% similarity to reference COI barcodes successfully assigned to higher taxonomic levels using a Bayesian phylogenetic approach implemented in the Statistical Assignment Package (SAP) and closest Blast hits (if >85% similarity, see Methods section).

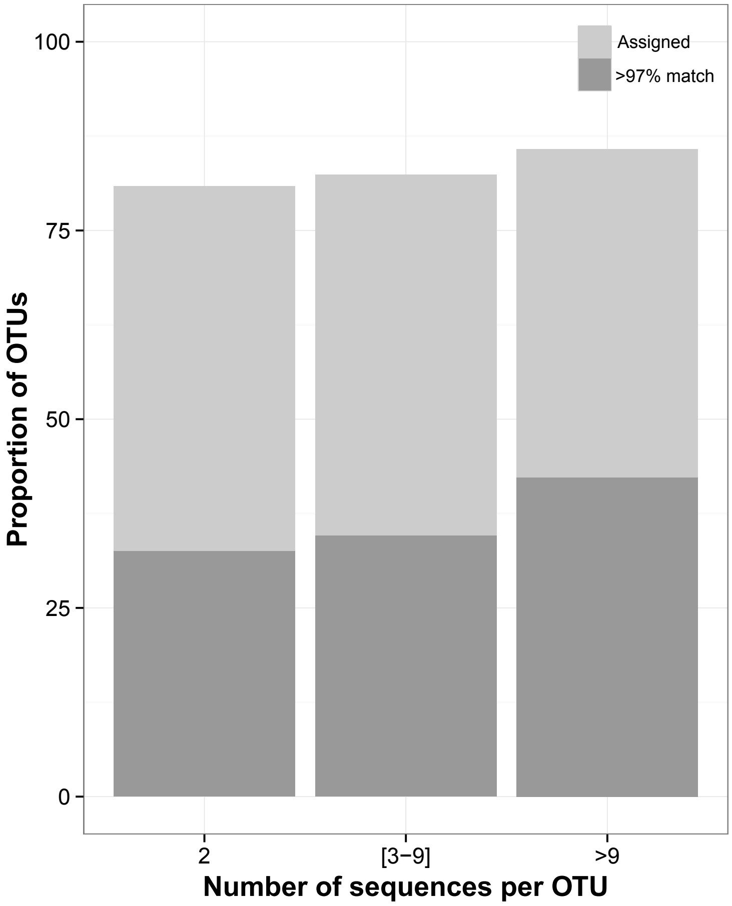

Figure S4 Relationship between number of OTUs and number of reads.

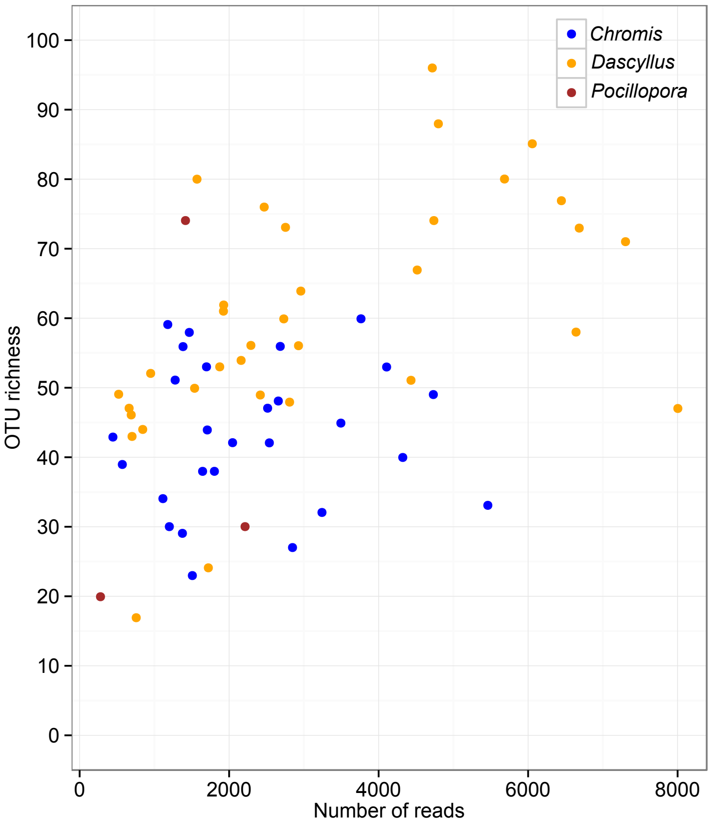

Figure S5 Robustness of ecological patterns to the loss of data during rarefaction. Non-metric multidimensional scaling plots were computed at two levels of rarefaction, 900 reads and 1200 reads, using Jaccard and Bray-Curtis metrics.

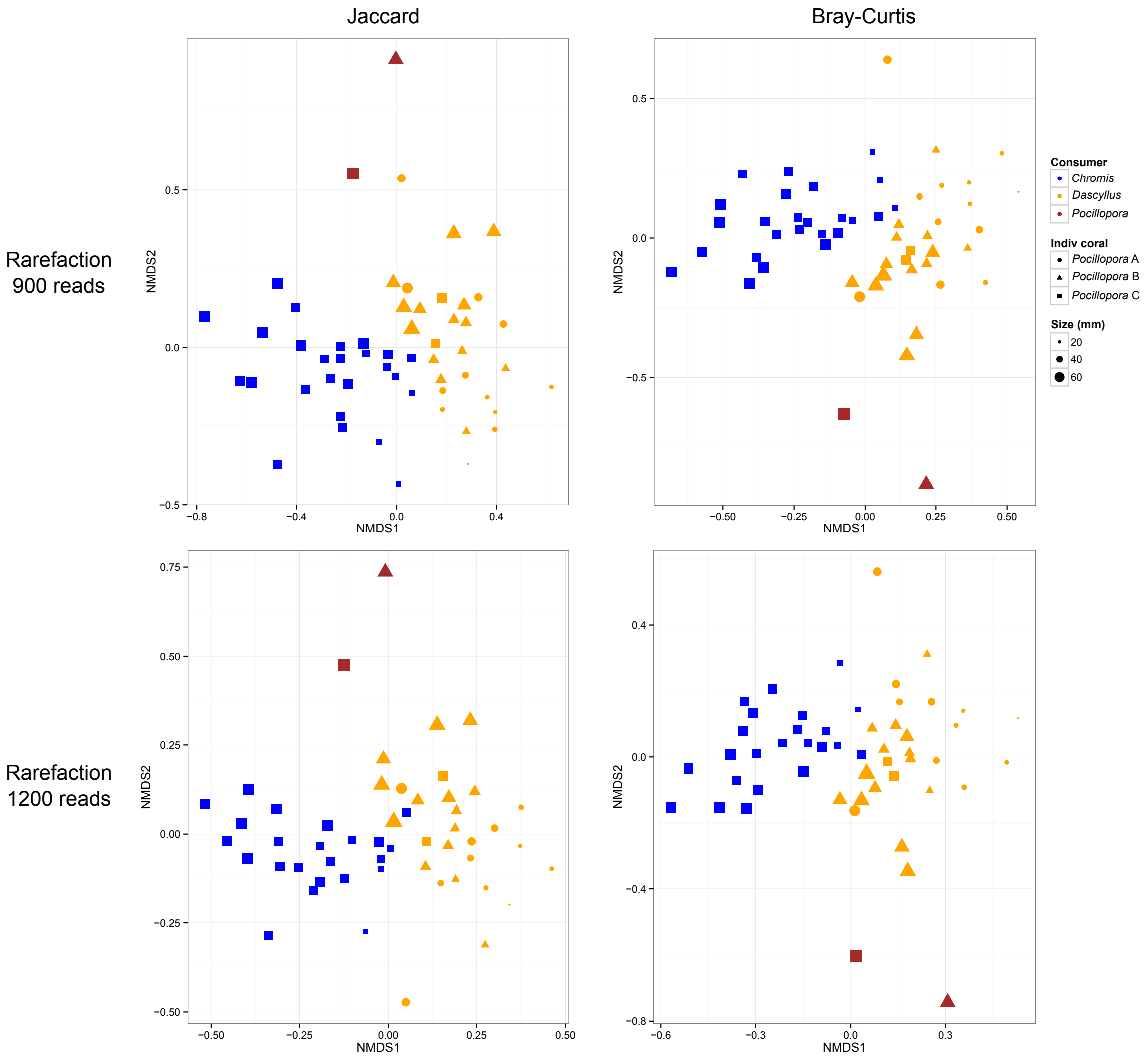

Table S1 Strategy used for multiplexing samples. Primer and 454 MID tag sequences can be found in Table S2. Raw sequence data files are available from Figshare (https://doi.org/10.6084/m9.figshare.5808618; https://doi.org/10.6084/m9.figshare.5808621) and the NCBI Short Read Archive (BioSample accessions: SAMN10780924-SAMN10780987).

Sample ID	Species	Coral host	Total length (mm)	Primer set	454 MID tag no.	454 run no.
DF.2155	*Dascyllus flavicaudus*	A	65	mlCOIint_Tag4-jgHCO_Tag4	RL6	1
DF.2165	*Dascyllus flavicaudus*	A	45	mlCOIint_Tag1-jgHCO_Tag1	RL5	1
DF.2167	*Dascyllus flavicaudus*	A	41	mlCOIint_Tag3-jgHCO_Tag3	RL4	2
DF.2173	*Dascyllus flavicaudus*	A	50	mlCOIint_Tag5-jgHCO_Tag5	RL4	2
DF.2278	*Dascyllus flavicaudus*	A	41	mlCOIint_Tag5-jgHCO_Tag5	RL5	2
DF.2280	*Dascyllus flavicaudus*	A	33	mlCOIint_Tag2-jgHCO_Tag2	RL6	2
DF.2281	*Dascyllus flavicaudus*	A	13	mlCOIint_Tag3-jgHCO_Tag3	RL6	2
DF.2282	*Dascyllus flavicaudus*	A	30	mlCOIint_Tag4-jgHCO_Tag4	RL4	1
DF.2283	*Dascyllus flavicaudus*	A	28	mlCOIint_Tag1-jgHCO_Tag1	RL4	1
DF.2284	*Dascyllus flavicaudus*	A	26	mlCOIint_Tag4-jgHCO_Tag4	RL6	2
DF.2285	*Dascyllus flavicaudus*	A	28	mlCOIint_Tag2-jgHCO_Tag2	RL4	1
DF.2286	*Dascyllus flavicaudus*	A	28	mlCOIint_Tag5-jgHCO_Tag5	RL6	2
DF.2290	*Dascyllus flavicaudus*	A	51	mlCOIint_Tag2-jgHCO_Tag2	RL7	2
Poc.A	*Pocillopora eydouxi*	A	N/A	mlCOIint_Tag6-jgHCO_Tag6	RL8	1
Poc.A	*Pocillopora eydouxi*	A	N/A	mlCOIint_Tag7-jgHCO_Tag7	RL8	1
DF.2157	*Dascyllus flavicaudus*	B	68	mlCOIint_Tag5-jgHCO_Tag5	RL3	2
DF.2158	*Dascyllus flavicaudus*	B	75	mlCOIint_Tag1-jgHCO_Tag1	RL4	2
DF.2159	*Dascyllus flavicaudus*	B	67	mlCOIint_Tag5-jgHCO_Tag5	RL6	1
DF.2162	*Dascyllus flavicaudus*	B	61	mlCOIint_Tag2-jgHCO_Tag2	RL4	2
DF.2166	*Dascyllus flavicaudus*	B	53	mlCOIint_Tag1-jgHCO_Tag1	RL6	1
DF.2168	*Dascyllus flavicaudus*	B	48	mlCOIint_Tag2-jgHCO_Tag2	RL5	1
DF.2169	*Dascyllus flavicaudus*	B	84	mlCOIint_Tag1-jgHCO_Tag1	RL7	1
DF.2170	*Dascyllus flavicaudus*	B	79	mlCOIint_Tag4-jgHCO_Tag4	RL4	2
DF.2171	*Dascyllus flavicaudus*	B	49	mlCOIint_Tag3-jgHCO_Tag3	RL5	1
DF.2172	*Dascyllus flavicaudus*	B	39	mlCOIint_Tag5-jgHCO_Tag5	RL4	1
DF.2174	*Dascyllus flavicaudus*	B	74	mlCOIint_Tag1-jgHCO_Tag1	RL5	2
DF.2175	*Dascyllus flavicaudus*	B	70	mlCOIint_Tag2-jgHCO_Tag2	RL5	2
DF.2271	*Dascyllus flavicaudus*	B	63	mlCOIint_Tag3-jgHCO_Tag3	RL6	1
DF.2273	*Dascyllus flavicaudus*	B	52	mlCOIint_Tag3-jgHCO_Tag3	RL5	2
DF.2274	*Dascyllus flavicaudus*	B	50	mlCOIint_Tag4-jgHCO_Tag4	RL5	1
DF.2276	*Dascyllus flavicaudus*	B	47	mlCOIint_Tag4-jgHCO_Tag4	RL5	2
DF.2279	*Dascyllus flavicaudus*	B	39	mlCOIint_Tag1-jgHCO_Tag1	RL6	2
DF.2289	*Dascyllus flavicaudus*	B	55	mlCOIint_Tag1-jgHCO_Tag1	RL7	2
Poc.B	*Pocillopora eydouxi*	B	N/A	mlCOIint_Tag1-jgHCO_Tag1	RL8	1
Poc.B	*Pocillopora eydouxi*	B	N/A	mlCOIint_Tag8-jgHCO_Tag8	RL8	1

CV.2133	*Chromis viridis*	C	63	mlCOIint_Tag4-jgHCO_Tag4	RL3	1
CV.2134	*Chromis viridis*	C	59	mlCOIint_Tag1-jgHCO_Tag1	RL1	2
CV.2135	*Chromis viridis*	C	43	mlCOIint_Tag2-jgHCO_Tag2	RL1	1
CV.2136	*Chromis viridis*	C	51	mlCOIint_Tag2-jgHCO_Tag2	RL1	2
CV.2137	*Chromis viridis*	C	52	mlCOIint_Tag2-jgHCO_Tag2	RL2	1
CV.2138	*Chromis viridis*	C	41	mlCOIint_Tag1-jgHCO_Tag1	RL1	1
CV.2139	*Chromis viridis*	C	44	mlCOIint_Tag3-jgHCO_Tag3	RL1	1
CV.2140	*Chromis viridis*	C	65	mlCOIint_Tag2-jgHCO_Tag2	RL3	1
CV.2142	*Chromis viridis*	C	51	mlCOIint_Tag3-jgHCO_Tag3	RL1	2
CV.2143	*Chromis viridis*	C	60	mlCOIint_Tag1-jgHCO_Tag1	RL3	1
CV.2144	*Chromis viridis*	C	52	mlCOIint_Tag1-jgHCO_Tag1	RL2	1
CV.2145	*Chromis viridis*	C	51	mlCOIint_Tag4-jgHCO_Tag4	RL1	2
CV.2146	*Chromis viridis*	C	35	mlCOIint_Tag5-jgHCO_Tag5	RL1	2
CV.2147	*Chromis viridis*	C	34	mlCOIint_Tag1-jgHCO_Tag1	RL2	2
CV.2148	*Chromis viridis*	C	31	mlCOIint_Tag2-jgHCO_Tag2	RL2	2
CV.2149	*Chromis viridis*	C	53	mlCOIint_Tag3-jgHCO_Tag3	RL2	2
CV.2150	*Chromis viridis*	C	61	mlCOIint_Tag4-jgHCO_Tag4	RL2	2
CV.2152	*Chromis viridis*	C	56	mlCOIint_Tag5-jgHCO_Tag5	RL2	2
CV.2153	*Chromis viridis*	C	47	mlCOIint_Tag5-jgHCO_Tag5	RL1	1
CV.2154	*Chromis viridis*	C	64	mlCOIint_Tag1-jgHCO_Tag1	RL3	2
CV.2293	*Chromis viridis*	C	49	mlCOIint_Tag2-jgHCO_Tag2	RL3	2
CV.2294	*Chromis viridis*	C	52	mlCOIint_Tag3-jgHCO_Tag3	RL2	1
CV.2295	*Chromis viridis*	C	64	mlCOIint_Tag3-jgHCO_Tag3	RL3	1
CV.2296	*Chromis viridis*	C	53	mlCOIint_Tag4-jgHCO_Tag4	RL2	1
CV.2297	*Chromis viridis*	C	45	mlCOIint_Tag4-jgHCO_Tag4	RL1	1
CV.2298	*Chromis viridis*	C	57	mlCOIint_Tag5-jgHCO_Tag5	RL2	1
CV.2300	*Chromis viridis*	C	58	mlCOIint_Tag3-jgHCO_Tag3	RL3	2
DF.2156	*Dascyllus flavicaudus*	C	57	mlCOIint_Tag4-jgHCO_Tag4	RL3	2
DF.2161	*Dascyllus flavicaudus*	C	52	mlCOIint_Tag5-jgHCO_Tag5	RL5	1
DF.2292	*Dascyllus flavicaudus*	C	29	mlCOIint_Tag3-jgHCO_Tag3	RL4	1
Poc.C	*Pocillopora eydouxi*	C	N/A	mlCOIint_Tag2-jgHCO_Tag2	RL8	1
Poc.C	*Pocillopora eydouxi*	C	N/A	mlCOIint_Tag3-jgHCO_Tag3	RL8	1

Table S2 List of primers and 454Multiplex Identifiers (MID tags) used in this study

Label	Sequence (5′–3′)
mlCOIint_Tag1	AGCACGGGWACWGGWTGAACWGTWTAYCCYCC
mlCOIint_Tag2	ACGCAGGGWACWGGWTGAACWGTWTAYCCYCC
mlCOIint_Tag3	ACTATCGGWACWGGWTGAACWGTWTAYCCYCC
mlCOIint_Tag4	AGACGCGGWACWGGWTGAACWGTWTAYCCYCC
mlCOIint_Tag5	ATCGACGGWACWGGWTGAACWGTWTAYCCYCC
mlCOIint_Tag6	TCTCTGGGWACWGGWTGAACWGTWTAYCCYCC
mlCOIint_Tag7	TGATCGGGWACWGGWTGAACWGTWTAYCCYCC
mlCOIint_Tag8	TAGTGCGGWACWGGWTGAACWGTWTAYCCYCC
jgHCO_Tag1	AGCACGTANACYTCNGGRTGNCCRAARAAYCA
jgHCO_Tag2	ACGCAGTANACYTCNGGRTGNCCRAARAAYCA
jgHCO_Tag3	ACTATCTANACYTCNGGRTGNCCRAARAAYCA
jgHCO_Tag4	AGACGCTANACYTCNGGRTGNCCRAARAAYCA
jgHCO_Tag5	ATCGACTANACYTCNGGRTGNCCRAARAAYCA
jgHCO_Tag6	TCTCTGTANACYTCNGGRTGNCCRAARAAYCA
jgHCO_Tag7	TGATCGTANACYTCNGGRTGNCCRAARAAYCA
jgHCO_Tag8	TAGTGCTANACYTCNGGRTGNCCRAARAAYCA
*C.viridis*_blocker	CAAAGAATCAGAATAGGTGTTGGTACAGA-C3
*D.flavicaudus*_blocker	CAAAGAATCAGAATAAATGTTGATAGAGG-C3
*Pocillopora*_blocker	CAAAGAATCAAAATAAATGCTGAAATAAT-C3
Roche MID RL1	"ACACGACGACT", "AGTCGTGGTGT"
Roche MID RL2	"ACACGTAGTAT", "ATACTAGGTGT"
Roche MID RL3	"ACACTACTCGT", "ACGAGTGGTGT"
Roche MID RL4	"ACGACACGTAT", "ATACGTGGCGT"
Roche MID RL5	"ACGAGTAGACT", "AGTCTACGCGT"
Roche MID RL6	"ACGCGTCTAGT", "ACTAGAGGCGT"
Roche MID RL7	"ACGTACACACT", "AGTGTGTGCGT"
Roche MID RL8	"ACGTACTGTGT", "ACACAGTGCGT"

Table S3 Permutational multivariate analysis (PERMANOVA) testing inter- and intra-specific differences in diet at two levels of rarefaction, 900 reads and 1200 reads, using Jaccard and Bray-Curtis. One out of the three *Pocillopora* samples (C) was discarded at both levels of rarefaction. Therefore, interspecific diet partitioning was tested between *Chromis* and *Dascyllus* only. Similarly, individual *Dascyllus* collected on *Pocillopora* C were not included in the PERMANOVA analyses because one out of the three samples was discarded after rarefaction.

	Beta	Source of variation	Df	SS	F Model	R^2	*P-value*
**A- Rarefaction 900 reads**							
Inter-specific	Jaccard	Species	1	1.500	4.6	0.08	<0.001***
(*Chromis* vs. *Dascyllus*)		Residuals	51	17.1		0.92	
	Bray-Curtis	Species	1	3.7	13.9	0.21	<0.001***
		Residuals	51	13.6		0.79	
Intra-specific	Jaccard	Coral host	1	0.5	1.5	0.06	0.003**
(*Dascyllus* on *Pocillopora* A vs. B)		Residuals	24	8		0.94	
	Bray-Curtis	Coral host	1	0.49	1.6	0.06	0.02**
		Residuals	24	7.5		0.94	
**B- Rarefaction 1200 reads**							
Inter-specific	Jaccard	Species	1	1.500	4.7	0.09	<0.001***
(*Chromis* vs. *Dascyllus*)		Residuals	48	15.7		0.91	
	Bray-Curtis	Species	1	3.6	13.8	0.22	<0.001***
		Residuals	48	12.6		0.78	
Intra-specific	Jaccard	Coral host	1	0.48	1.4	0.06	0.002**
(*Dascyllus* on *Pocillopora* A vs. B)		Residuals	23	7.66		0.94	
	Bray-Curtis	Coral host	1	0.45	1.4	0.06	0.05*
		Residuals	23	7.23		0.94	
